# Balloon-expandable transcatheter heart valve deformation: a rare complication during transcatheter aortic valve replacement

**DOI:** 10.1093/ehjcr/ytae285

**Published:** 2024-06-06

**Authors:** Akiko Masumoto, Hiroyuki Yamamoto, Nobuyuki Takahashi, Tomofumi Takaya

**Affiliations:** Division of Cardiovascular Medicine, Department of Internal Medicine, Hyogo Prefectural Harima-Himeji General Medical Center, Himeji 670-8560, Japan; Division of Cardiovascular Medicine, Department of Internal Medicine, Hyogo Prefectural Harima-Himeji General Medical Center, Himeji 670-8560, Japan; Division of Cardiovascular Medicine, Department of Internal Medicine, Hyogo Prefectural Harima-Himeji General Medical Center, Himeji 670-8560, Japan; Division of Cardiovascular Medicine, Department of Internal Medicine, Hyogo Prefectural Harima-Himeji General Medical Center, Himeji 670-8560, Japan; Department of Exploratory and Advanced Search in Cardiology, Kobe University Graduate School of Medicine, Kobe, Japan

An 80-year-old woman with a history of pacemaker implantation and reduced left ventricular function (left ventricular ejection fraction: 33%) presented with severe aortic stenosis. Computed tomography (CT) revealed stenoses of the bilateral iliac arteries and calcified abdominal aorta (*Figure 1A* and *B*). However, neither trans-subclavian nor direct aortic transcatheter aortic valve replacement (TAVR) was feasible due to subclavian artery stenosis and a porcelain ascending aorta. Moreover, the surgical risks were high, with a mortality risk for surgical aortic valve replacement, as measured by the Society of Thoracic Surgeons score, being 11.8%. After discussing the choice of valve system within the heart team, transfemoral TAVR with a balloon-expandable valve (BEV) was performed. This decision was made considering that 14 Fr eSheaths of the balloon-expandable systems would be suitable to pass through calcified or tortuous femoral arteries, compared to 14 Fr equivalent in-line sheaths of self-expandable systems. Intravascular lithotripsy was not available at the time of the procedure. Upon reaching the descending thoracic aorta with the delivery system, an outward bending of the transcatheter heart valve (THV) stent frame was observed, encountering slight resistance (*Figure 1C*). Moreover, balloon angioplasty using a 7 mm balloon aligned next to the THV failed to fix the deformation (*Figure 1D*). The delivery system could not be retracted into the sheath. In this case, retracting the THV system as a whole posed risks of further damaging heavily calcified arteries. Additionally, the heart team cautiously decided to proceed with deploying the valve while preparing surgical backup in case of rupture. The valve was advanced while protecting the deformed part with a balloon to prevent friction against the aortic intima: ‘balloon cushion technique’. The deformed part was directed towards the non-coronary cusp (NCC) in the ‘NCC isolation view’ on fluoroscopy, minimizing the risk of annular rupture (*Figure 1E*). The BEV was deployed with slow inflation and an underfilling of 3 mL, resulting in no signs of annular rupture or ventricular septal ruptures (*Figure 1F*; Supplementary material online, *Video S1*). Localized dissection was observed in the abdominal aorta, which was treated with endovascular stent grafts (*Figure 1G*). Subsequently, post-procedural CT confirmed no annular rupture (*Figure 1H*).

**Figure 1:**
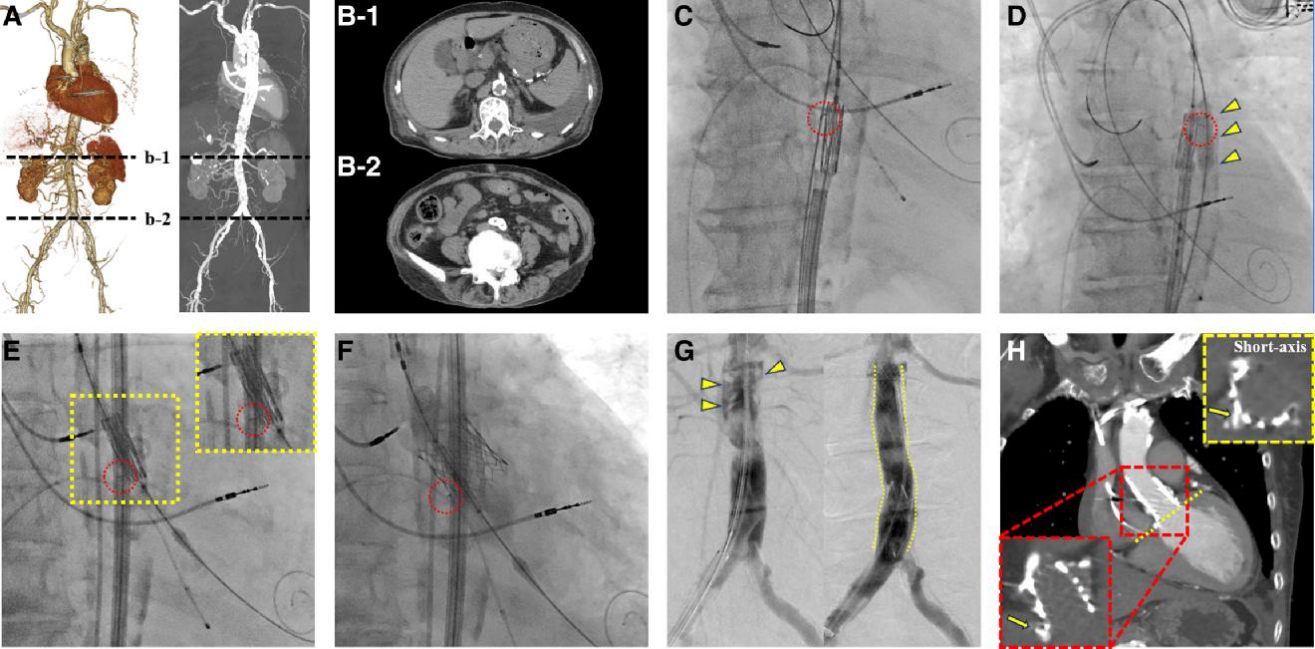
(*A*) Three-dimensional computed tomography (CT) angiography of transfemoral access. Abdominal aorta (*B*-1) and bilateral proximal iliac artery stenoses (*B*-2) with calcified plaque. (*C*) An outward bending of the stent frame (circle). (*D*) Balloon angioplasty (arrowheads) advanced via the contralateral femoral artery fails to fix the deformation (dotted circle). (*E*) Before and (*F*) after the deployment of the deformed valve (circle). (*G*) The angiogram after the valve deployment shows localized dissection in the abdominal aorta (arrowheads) for which endovascular stent grafts (dotted lines) are deployed for successful sealing. (*H*) CT scan after the procedure shows deformed valve (arrows) without any signs of aortic annular rupture.

Deformation of the BEV is extremely rare, which, in this case, may have occurred when the system passed through the severely calcified abdominal aorta. The THV needs to be inevitably deployed when advanced beyond the sheath, albeit with caution due to the risk of annular rupture associated with deploying such deformed valves. It is important not to exert excessive force on the system as the valve navigates through calcified anatomies. If encountered, the safety of the process could be enhanced by protecting the deformation with another balloon as a cushion against the aorta and deploying the valve with underfilling while having a full surgical backup. It is presumed that the risk of rupture for the protruding strut is lower when it faces the NCC side.^1^

## Lead author biography


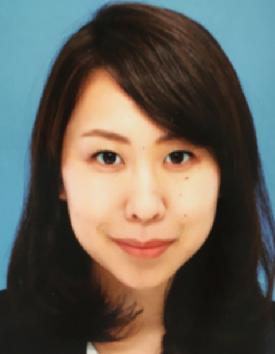


Akiko Masumoto obtained her license to practice medicine at Osaka University, Japan. After completing residency at the Kobe City Medical Center General Hospital, she enrolled as a structural heart disease fellow in cardiovascular medicine at the Hyogo Prefectural Harima-Himeji General Medical Center, where she works currently. She specializes in structural heart diseases and echocardiography.

## Supplementary Material

ytae285_Supplementary_Data

## Data Availability

The data underlying this article are available in the article and its online Supplementary material.

## Supplementary material

**Consent**: The authors confirm that written consent for submission and publication of this case report including images and associated text has been obtained from the patient in line with COPE guidance.

**Funding:** None.
